# Supplementary figures and images for: DNA methylome and transcriptome analysis established a model of four differentially methylated positions (DMPs) as a diagnostic marker in esophageal adenocarcinoma early detection
Source: PeerJ. 2021 May 7;9:e11355. doi: 10.7717/peerj.11355 (PMC8109010; doi:10.7717/peerj.11355)

A

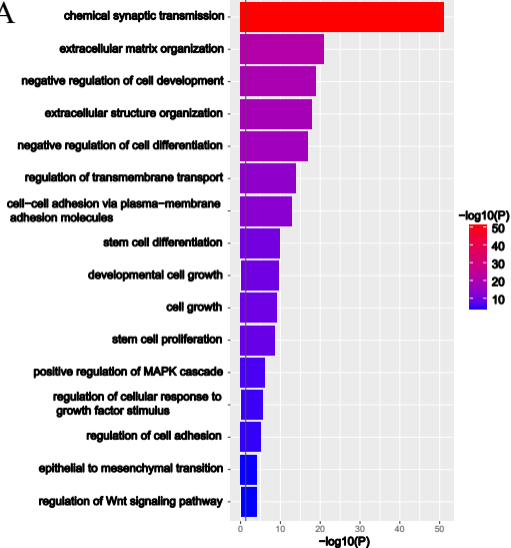

B

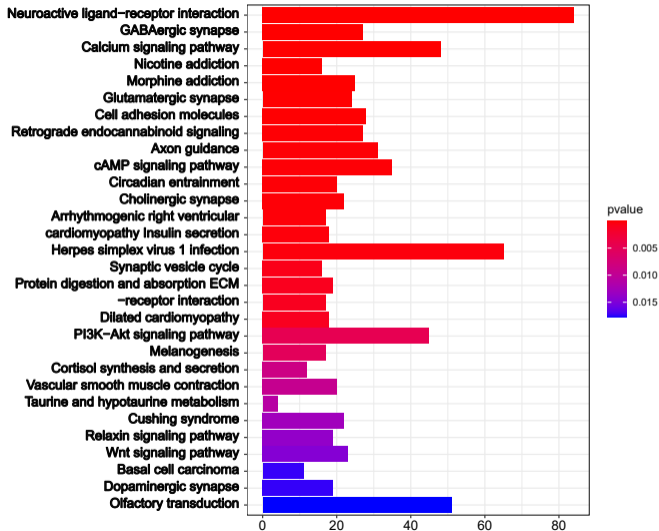

Supplement: Supplemental Information 4 — (A) GO analysis of aberrantly methylated genes. (B) KEGG pathway analysis of aberrantly methylated genes. [file peerj-09-11355-s004.pdf]

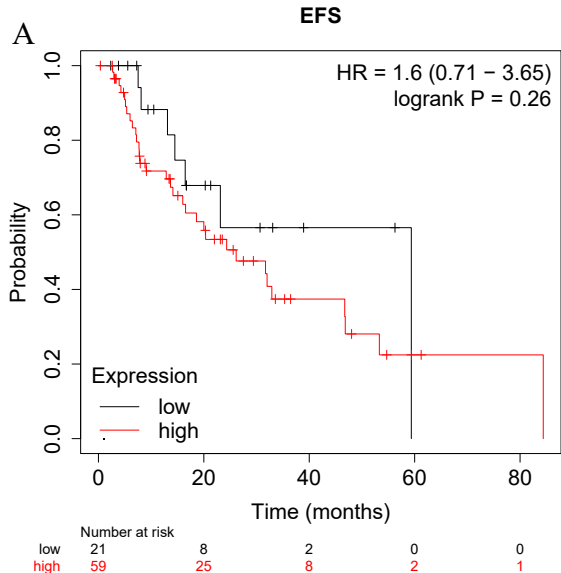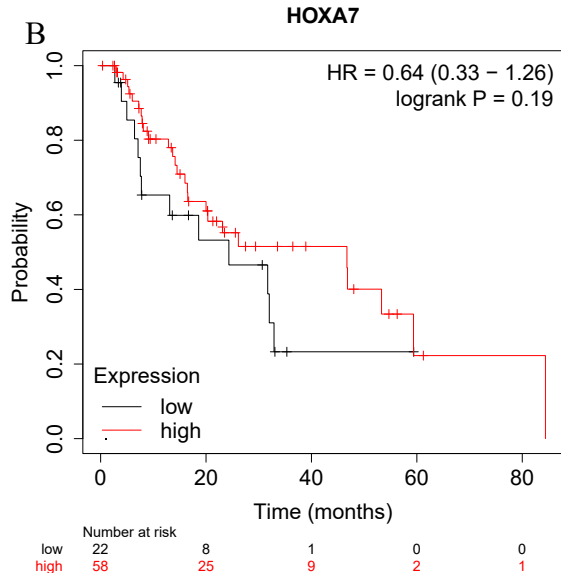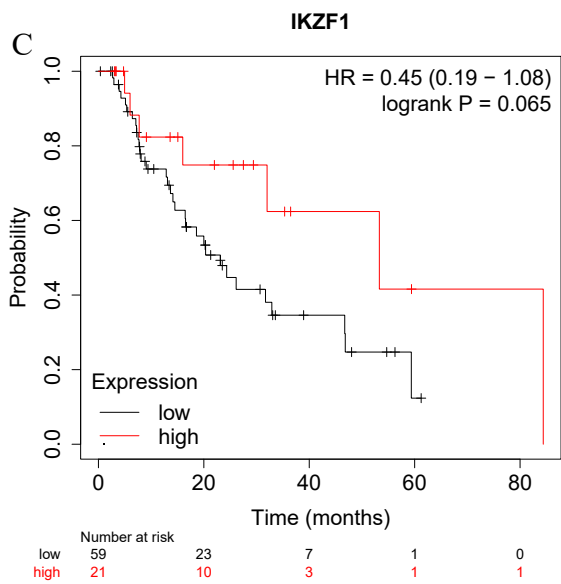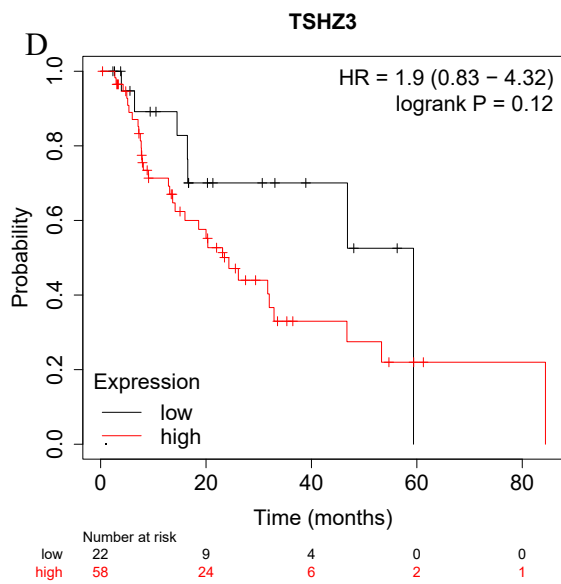

Supplement: Supplemental Information 5 — Kaplan Meier curves of (A) EFS (B) HOXA7 (C) IKZF1 (D) TSHZ3 for overall survival rates based on TCGA EAC samples. [file peerj-09-11355-s005.pdf]

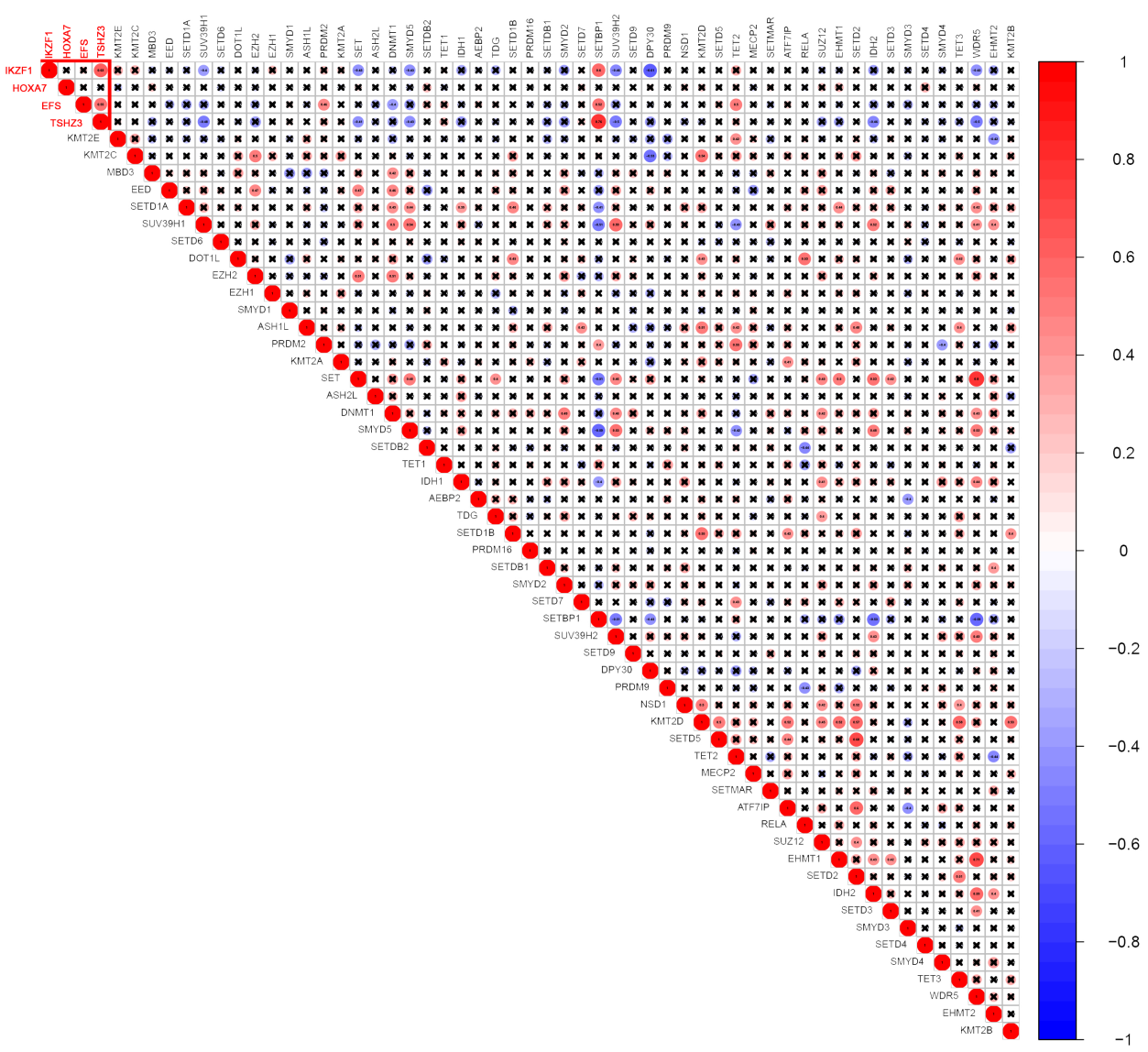

Supplement: Supplemental Information 6 — Correlation between known mediators of the CpG methylation and four genes [file peerj-09-11355-s006.pdf]

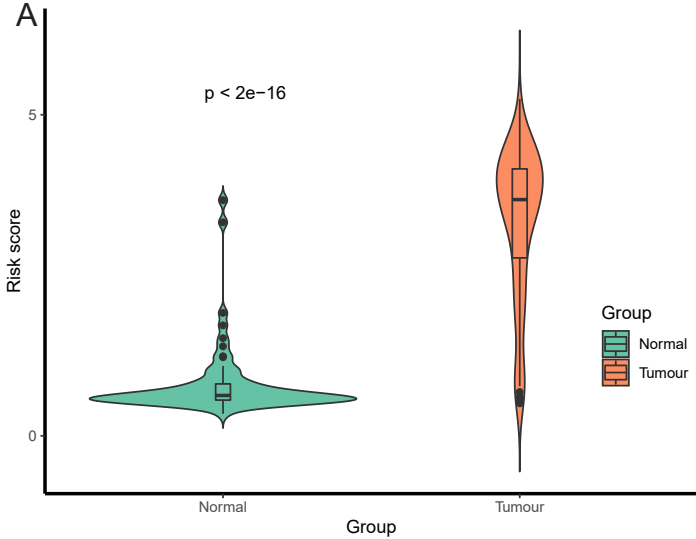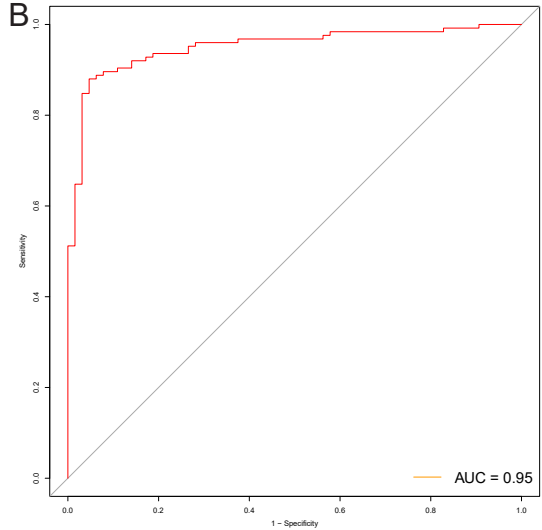

Supplement: Supplemental Information 7 — (A) Risk score of EAC tumor and normal tissues in GSE72872. (B) ROC curve of the diagnostic model in GSE72872. [file peerj-09-11355-s007.pdf]

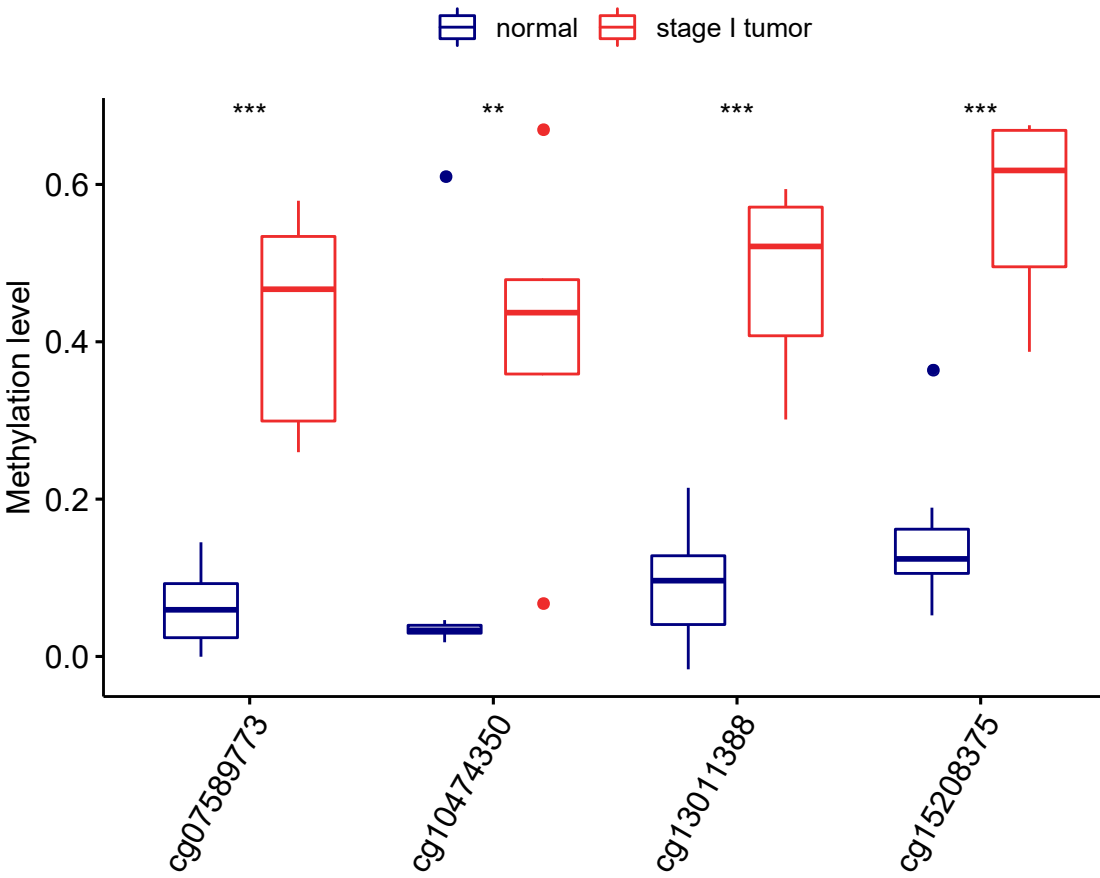

Supplement: Supplemental Information 8 — Methylation levels of the four CpGs between EAC tumor and normal tissues in GSE89181. [file peerj-09-11355-s008.pdf]
